# Direct Meta-Analyses Reveal Unexpected Microbial Life in the Highly Radioactive Water of an Operating Nuclear Reactor Core

**DOI:** 10.3390/microorganisms8121857

**Published:** 2020-11-25

**Authors:** Pauline C. M. Petit, Olivier Pible, Valérie Van Eesbeeck, Claude Alban, Gérard Steinmetz, Mohamed Mysara, Pieter Monsieurs, Jean Armengaud, Corinne Rivasseau

**Affiliations:** 1 Commissariat à l’Energie Atomique et aux Energies Alternatives (CEA), CNRS, INRAE, Université Grenoble Alpes, F-38054 Grenoble, France; petitpauline973@gmail.com (P.C.M.P.); claude.alban@cea.fr (C.A.); 2 Département Médicaments et Technologies pour la Santé (DMTS), CEA, INRAE, SPI, Université Paris-Saclay, F-30200 Bagnols-sur-Cèze, France; olivier.pible@cea.fr (O.P.); steinmetz.gerard@wanadoo.fr (G.S.); jean.armengaud@cea.fr (J.A.); 3 Microbiology Unit, The Belgian Nuclear Research Centre (SCK•CEN), Boeretang 200, B-2400 Mol, Belgium; valerie.van.eesbeeck@sckcen.be (V.V.E.); mohamed.mysara@sckcen.be (M.M.); pmonsieurs@itg.be (P.M.)

**Keywords:** environmental microbiome, nuclear reactor, irradiation, metabarcoding, 16S rRNA amplicon sequencing, proteotyping

## Abstract

The pools of nuclear reactor facilities constitute harsh environments for life, bathed with ionizing radiation, filled with demineralized water and containing toxic radioactive elements. The very few studies published to date have explored water pools used to store spent nuclear fuels. Due to access restrictions and strong handling constraints related to the high radioactivity level, nothing is presently known about life in water pools that directly cool nuclear cores. In this work, we investigated the microbial communities in the cooling pool of the French Osiris nuclear reactor using direct meta-omics approaches, namely, DNA metabarcoding and proteotyping based on 16S ribosomal RNA gene sequencing and on peptide analysis, respectively. We identified 25 genera in the highly radioactive core water supply during operation with radionuclide activity higher than 3 × 10^9^ Bq/m^3^. The prevailing genera *Variovorax* and *Sphingomonas* at operation were supplanted by *Methylobacterium*, *Asanoa*, and *Streptomyces* during shutdown. *Variovorax* might use dihydrogen produced by water radiolysis as an energy source.

## 1. Introduction

Nuclear reactor facilities constitute extreme environments for life and little information is available on the species capable of surviving in there. The few studies published to date on this type of environment have focused on pools used to store spent nuclear fuels once the fuel has been used for energy production. These water coolant pools sustain high levels of ionizing radiation originating from the cooling fuels, contain radioactive elements in solution including toxic metals, and are filled with demineralized water. The very few studies investigating the microbial diversity in such environments highlighted the presence of Proteobacteria, Firmicutes, Actinobacteria, Deinococcus-Thermus, and also a Chlorophyta [1,2,3,4]. These organisms represent a unique opportunity to study the mechanisms of radioresistance and radionuclide accumulation and to develop new decontamination biotechnologies [5]. Nuclear reactor installations also primarily house the reactor core, which is bathed by the primary cooling circuit. Due to the access restrictions and the difficulty in handling such samples, nothing is currently known about life in water used to cool nuclear reactor cores. Such an environment is subject to even more severe conditions than spent nuclear fuel pools (SNFPs) since the water directly flows around the fuel rods, sustaining enormous amounts of radiation and exhibiting extremely large radionuclide concentrations during reactor operation. In this work, we investigated an open-core reactor, the French Osiris reactor, in which the reactor block is immersed in a pool of water and in communication with it. We explored the microbial communities present in the Osiris reactor core coolant water during operation and compared them with those present at shutdown.

Most studies focusing on spent nuclear fuel storage pools have identified microorganisms after cultivation [1,2,3,4], which results in an important loss in diversity since only 0.1 to 10% of the microorganisms present in water are culturable [6]. Direct meta-analysis based on 16S ribosomal RNA (rRNA) gene sequencing (metabarcoding) is a powerful tool to explore microbial community composition in the environment [7]; however, several sources of biases, including, among others, DNA extraction, PCR amplification, the choice of the 16S rRNA primers and the size of the fragment to sequence, make this approach more qualitative than quantitative [8]. Metaproteomics based on peptide analysis by tandem mass spectrometry has been used to assess functional aspects of communities in their environment. Such analysis also provides taxonomical information on microbial communities, based on proteotyping [9], and estimates biomass contributions of each taxon with relatively good confidence [10], but has never been applied to samples from nuclear facilities. In this work, we investigated the microbial population present in the reactor’s pool using complementary direct approaches of metabarcoding and proteotyping.

## 2. Materials and Methods

### 2.1. Water Samples from the Cooling Pool of the Nuclear Reactor Core

**Figure 1:**
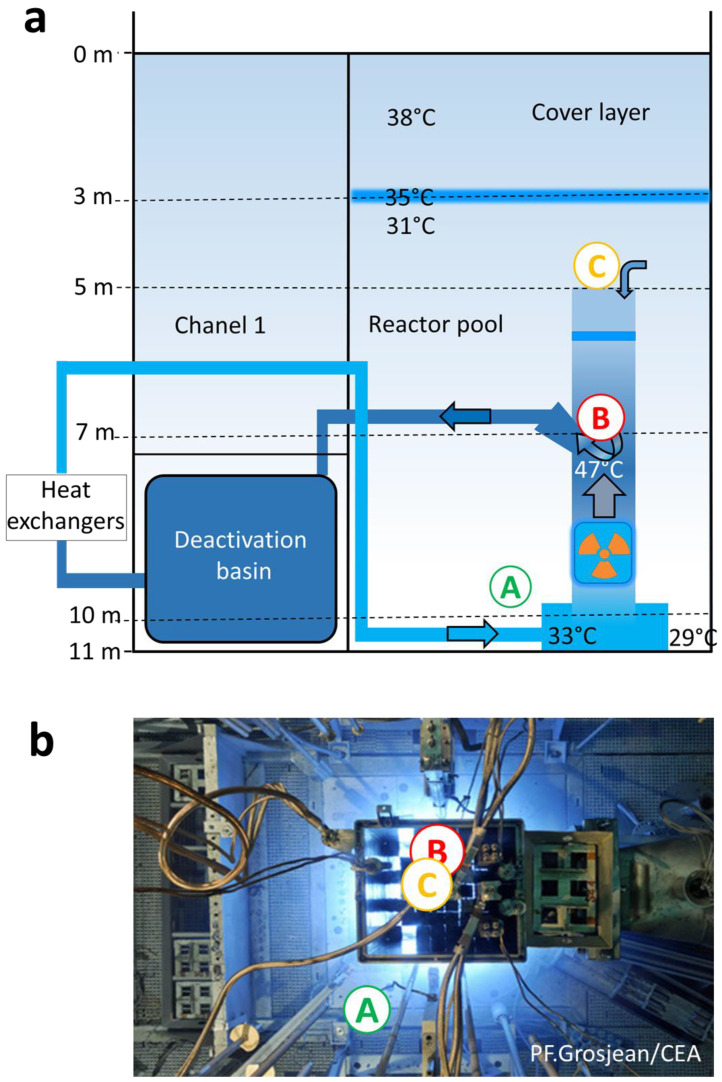
Configuration of the Osiris reactor’s pool (adapted from [11]) and sampling points A (pool’s bottom), B (inside the core unit), and C (above the core unit). (**a**) Schematic view, (**b**) top view. At shutdown, the radiation dose rate reached 25 Gy/h at point B, 15 µGy/h at point C, and was below 0.1 µGy/h at point A.

The facility studied is the French 70 MW nuclear reactor Osiris located in CEA Saclay. We investigated the cooling pool containing light water in direct contact with the reactor core. The reactor contains uranium sources inside the core unit which helps to position the fuel elements and channels the cooling water (Figure 1a). The pool is 11 m deep, 7.5 m long, and 6.5 m wide. Water samples were collected at three points of the basin: location A (Loc. A, bottom of the pool, 2 m away from the core), location B (Loc. B, inside the core unit), and location C (Loc. C, above the core unit). While the reactor was in operation, 2 L of water were collected at Loc. A on December 2015 using a permanently installed pipe extensively purged before sampling. The samples were collected directly into a sterile plastic container connected to the pipe. When the reactor was shut down, 8 L of water were collected on March 2017 at Loc. A using the same device, and at Loc. B and Loc. C using a sterile sampling vial (Wildco, Yulee, FL, USA) attached to a pole which was filled underwater at the sampling point. Comparison of the microbial composition of samples collected by both methods validated the sampling methodology and brought out that either sampling mode did not induce unexpected contamination.

While in operation, the pool’s demineralized water, pH 7.0, conductivity 0.5 µS/cm, maintained at 30 ± 1 °C below a 3.5 m depth, contained the β-emitter ^3^H (2.9 × 10^8^ Bq/m^3^) and various γ-emitter radionuclides detailed in Table S1 yielding a total gamma activity of 3.2 × 10^9^ Bq/m^3^. During the shutdown, the pool’s demineralized water, pH 5.4, conductivity 0.5 µS/cm, contained the β-emitter ^3^H (2.5 × 10^8^ Bq/m^3^) and various γ-emitters detailed in Table S1 yielding a total gamma activity of 3.3 × 10^6^ Bq/m^3^. During the cycle, the water was continuously filtered at 500 m^3^/h and purified on ion exchange resins at 30 m^3^/h, with a turnover of 18 h. The 500 m^3^/h filtration operation was maintained at shutdown. Microbial cell density was assessed using a Malassez cell after water centrifugation (50 mL) at 20,000× *g* for 40 min.

Samples collected during the reactor operation were stored in the sterile containers for three days at 4 °C before handling to reduce their activity by radioactive decay of ^24^Na (half-life 15 h) to 24 µS/h in contact with water. Microorganisms were then concentrated and harvested either by centrifugation at 16,000× *g* for 20 min at 10 °C or by filtration on 0.22 µm sterile polyethersulfone filters (MicroFunnel, Pall Corporation, Saint-Germain-en-Laye, France) in a sterile environment. Samples harvested during shutdown were processed immediately after collection. Each sample was divided into three 1 L aliquots for metabarcoding and 4.5 L for proteotyping analysis. They were then centrifuged or filtered as above. During this pre-treatment step by centrifugation or filtration, contamination controls were carried out by using tubes containing liquid culture media (LB, BHI, and TSB diluted ten times, R2A and NB diluted twice) which remained negative. The way potential contamination was challenged throughout sample treatment is detailed in Supplementary materials Table S4. The sample concentration step has been introduced to ensure that biomass in all subsequent sample treatment steps was sufficient for an exhaustive analysis of the population and to avoid issues arising with low-biomass samples [12,13]. These concentrated biomass samples, which contain of the order of 10^6^ cells (see the Results and Discussion section for the cell density values), were then treated for DNA extraction and sequencing and for protein extraction and peptide analysis.

### 2.2. DNA Extraction, Illumina Sequencing, and Sequence Data Analysis by Metabarcoding

Three methods combining different concentration protocols and DNA extraction methods were used to treat the 1 L water samples prior to amplicon sequencing. The use of different protocols enabled us to assess potential exogenous contamination originating from processing methods or DNA reagents. These three methods included centrifugation at 16,000× *g* for 20 min followed by phenol–chloroform–isoamyl alcohol DNA extraction, adapted from Vilchez-Vargas [14] (Supplementary method) (Method 1), filtration on 0.22 µm filter followed by phenol–chloroform–isoamyl alcohol DNA extraction (Method 2), and filtration on 0.22 µm filter followed by DNA extraction using the DNeasy PowerWater kit (Qiagen, Hilden, Germany) (Method 3). Methods 1 and 2 were used in the 2015 campaign. The three methods were used in the 2017 campaign. 

Control samples and samples of different origin were processed alongside the Osiris samples through pretreatment, DNA extraction, amplification, library preparation, sequencing, and data treatment. A mock ZymoBIOMICS (Zymo Research, Irvine, CA, USA) DNA standard was also processed concomitantly from the amplification stage (Table S4).

The V4V5 region of the 16S rRNA gene was amplified by LGC Genomics, UK, using the primers pair 515YF-926R (5′-GTGYCAGCMGCCGCGGTAA-3′ and 5′-CCGYCAATTYMTTTRAGTTT-3′) [15] and the resulting amplicons were sequenced on an Illumina MiSeq platform using V3 chemistry (2 × 300 bp) (Supplementary method). Sequencing data were processed through the OCToPUS (version 1) pipeline (Belgian nuclear research center/Vrije Universiteit Brussel/KULeuven, Mol, Brussels, Leuven, Belgium) [16] for contig assembly, quality filtering, de-noising, chimera removal, and operational taxonomic unit (OTU) clustering at 97% cutoff. Each OTU was taxonomically assigned at 80% confidence score against the Ribosomal Database Project database (version 16) using mothur (version 1.39) software (Michigan University, Ann Arbor, MI, USA) [17]. To improve taxonomic assignment, the main OTUs unidentified at the genus level were blasted against the nucleotide collection database using Megablast in the Nucleotide BLAST (version 2.8.1) tool from NCBI (National Center for Biotechnology Information/National Institutes of Health, Rockville Pike, Bethesda, MD, USA). Based on the ZymoBIOMICS standard analysis, only OTUs whose abundance exceeded a cut-off threshold of 0.016% were considered, to minimize false-positives rate (Table S5). Alpha diversity was calculated using mothur. For sample comparison and mean value calculation, sequencing results were subsampled at the second lowest read count (28,977 reads) using mothur.

### 2.3. Protein Extraction, Peptide Analysis Using nanoLC–MS/MS, and Taxonomic Analysis by Proteotyping

Following filtration, microorganisms were resuspended in 15 mL of sterile MilliQ water, centrifuged at 20,000× *g* for 20 min at 10 °C, and processed as described [18]. After addition of 50 µL of Laemmli buffer LDS1X (Invitrogen, Illkirch, France) and bead-beating, protein samples were denatured for 5 min at 99 °C and purified by electrophoresis on 4–12% gradient NuPAGE gels (Invitrogen). The whole proteome band was recovered, reduced with dithiothreitol, treated with iodoacetamide, and proteolyzed with trypsin (Gold Mass Spectrometry Grade, Promega, Walldorf, Germany) in the presence of 0.01% ProteaseMAX (Promega) [19]. Peptides were analyzed using a Q Exactive HF mass spectrometer (Thermo Scientific, Waltham, MA, USA) equipped with an ultra-high field Orbitrap analyzer and coupled to an Ultimate 3000 176 RSL Nano LC System (Thermo). They were separated on an Acclaim PepMap 100 C18 column using a 60 min gradient of CH_3_CN in 0.1% formic acid [20]. The Q Exactive HF instrument was operated with a Top20 strategy with MS/MS on ions potentially charged +2 or +3 and a 10 s dynamic exclusion. MS/MS spectra were interpreted with the National Center for Biotechnology Information non-redundant fasta file (NCBI-nr) database with the Mascot (version 2.6) search engine (Matrix Science, London, UK). Peptide to MS/MS spectrum assignation was performed with full trypsin specificity, a maximum of one missed cleavage, mass tolerances of 5 ppm on the parent ion and 0.02 Da on the MS/MS, static modification of carboxyamidomethylated cysteine (+57.0215), and oxidized methionine (+15.9949) as dynamic modification. Mascot data files were post-processed to obtain taxonomical proteotyping based on peptide-to-taxa assignments. A positive control consisting in a Zymobiomics mixture of ten microorganisms was treated in similar conditions and the results have been recently published [21].

## 3. Results and Discussion

**Figure 2:**
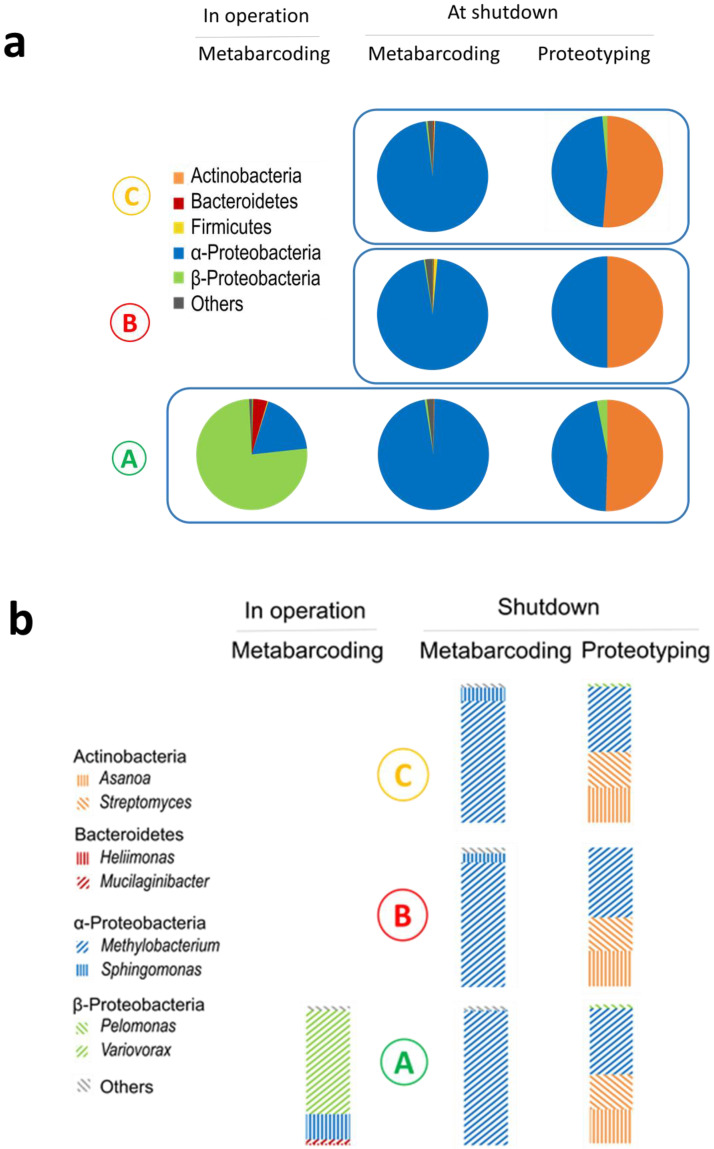
Microbial communities present in the cooling pool of the Osiris nuclear reactor core. (**a**) Main phyla (class for Proteobacteria) and (**b**) main genera (abundance above 1%) detected in operation and at shutdown using metabarcoding and proteotyping. Mean taxon abundance obtained by the different methods (see Materials and Methods Section).

During the reactor working cycle in December 2015, the core’s pool water was highly radioactive, with a tritium activity reaching 2.9 × 10^8^ Bq/m^3^ and a gamma activity reaching 3.3 × 10^9^ Bq/m^3^, mainly due to radioactive sodium (^24^Na, Table S1). The concentration of gamma-emitters was 1000 to 10,000 times higher than that prevailing in the SNFPs, such as in the one previously investigated by our team [5]. For security reasons, water was only sampled at the bottom of the pool (Loc. A) and not in the core unit (Figure 1a,b). The samples were stored for three days to reduce their radioactivity to a level enabling their handling in controlled conditions. Metabarcoding analyses yielded more than 50,000 raw reads (Table S2). The microbial cell density was below 0.5 × 10^3^ cells/mL which is consistent with the quantity of DNA extracted. The rarefaction curves reached an asymptote, indicating sufficient sequencing depth (Figure S1). The sample alpha diversity quantified by the Shannon index, which combines species richness and evenness, was below 1. A single OTU, classified as Comamonadaceae family and attributed to *Variovorax* sp. using BLASTn (top hits at 100% similarity), dominated the population. As shown in Figure 2a, Proteobacteria were largely represented (95% of the sequences) with 16 out of the 25 identified genera (Figure S2). The two most represented genera were *Variovorax* (75%) and *Sphingomonas* (18%) (Figure 2b).

During the reactor shutdown in March 2017, the tritium activity was near to that in operation whereas the gamma activity was 1000 times lower. Water could be sampled inside and above the core unit (Loc. B and Loc. C, Figure 1a) where the radiation dose rate reached 25 Gy/h and 15 µGy/h, respectively, as well as from Loc. A where the dose rate was below 0.1 µGy/h. Sampling at three different locations corresponding to very different irradiation dose rates, by a factor of one hundred million, and different water circulation areas enabled us to test the homogeneity of the microbial composition and concentration in the pool. The microbial cell density reached 2.7 ± 0.6 × 10^3^ cells/mL, 0.5 ± 0.2 × 10^3^ cells/mL, and 1.4 ± 0.2 × 10^3^ cells/mL at Loc. A, B, and C, respectively. The continuous water filtering and cleaning at a high flow rate, the low conductivity, and the high radiation dose rate maintained a low microbial density, similar to that reported in some SNFPs [2,3]. Though less dense, bacteria were not so rare at Loc. B where they sustained 25 Gy/h. With a single exception, 29,000 to 127,000 sequences were obtained. Rarefaction curves reached an asymptote. The alpha diversity was still low (Shannon index 0.2–1.2) (Table S2, Figure S1). Overall, 90 OTUs were identified. This is considerably below the several thousands of OTUs detected using metabarcoding in an SNFP where much less stringent conditions yielded higher microbial density and diversity (1.8 × 10^3^ to 2.8 × 10^5^ cell/mL, Shannon index of 5) [22]. Proteobacteria were predominant (97% of the sequences) (Figure 2a), comprising the genera *Methylobacterium* prevailing in all locations (one OTU, 87–97% depending on the sampling point) and *Sphingomonas* mostly present at Loc. B and Loc. C (0.1–9%) (Figure 2b, Figures S3–S5). Major OTUs representing 98.8% of the sequences were common to the three locations (Figure S6).

The microbial composition of the samples obtained at shutdown, which were rich in biological material, could be determined by proteotyping by tandem mass spectrometry (Table S3). Proteotyping data highlighted the predominance of two phyla in equal proportion irrespective of the sampling point, namely, Proteobacteria with the genus *Methylobacterium* (50% of the taxon-spectrum matches) and Actinobacteria with two genera *Asanoa* (25%) and *Streptomyces* (25%) (Figure 2a,b). The main genera were common to all locations likely due to the continuous and intense water circulation despite the stronger selection pressure at Loc. B.

The DNA metabarcoding and proteotyping approaches were highly complementary and both highlighted *Methylobacterium* as the most abundant genus. By contrast, the other two main genera of the Actinobacteria phylum were uniquely detected by proteotyping. Proteotyping can be applied on any biological material, dead or alive [23,24]. The protocol used does not introduce specific bias depending on the Gram staining. Conversely, the metabarcoding approach suffers from non-quantitative representativeness, particularly for Gram-positive bacteria. Most classical DNA extraction protocols considerably underestimate this fraction, likely due to the higher mechanical resistance of the bacterial cell walls [25]. Proteotyping with shotgun tandem mass spectrometry data probably gives a more accurate assessment of the biomass contributions of the main members of the consortia, despite biases linked to protein extraction, cell size, and a less extensive database [10]. Nonetheless, the higher sensitivity of metabarcoding brought to light low abundance species and diversity.

It should be highlighted that the species detected here are only prokaryotes. Although analysis of the 18S rRNA gene sequence was not performed in the present study, which focuses on the identification of bacteria, proteotyping could have allowed the detection of eukaryotic microbes. As recently shown, this approach can actually detect in the same run prokaryotes and eukaryotes such as fungi and microalgae [23]. In the present results, however, no specific peptides were found for any eukaryotic unicellular organisms.

Our data show that life was present in the cooling pool of an operational nuclear reactor, albeit with a restricted number of bacteria prevailing in this environment. The surface of the pool is open to the ambient air, and loading and unloading operations are carried out within the pool. Consequently, these environmental bacteria might have gained entry to the pool through airborne or equipment contamination owing to the traffic in and out of the reactor and to the operations of maintenance and exploitation. The strong selection pressure implies that the microorganisms detected in this pool might have been specifically selected for. 

It is likely that at least part of the microbial community was alive in the reactor pool water because it was actually able to multiply in growth conditions. Indeed, aliquots of water samples cultured in different media showed biomass growth after seven days [26]. Proteotyping analysis of the consortia in the culture highlighted the presence of genera identified by the direct approaches described here, including notably *Variovorax*, *Sphingomonas*, and *Methylobacterium* [26]. Another point supporting the hypothesis of the survival of these bacteria in the radiative environment is the presence of the same genera in both campaigns. This could imply that these microorganisms can be metabolically active under such conditions. 

Their presence in suspension in water despite the continuous circulation and filtering either may be linked to the heterogeneity of water mixing which, in certain areas, could allow space and time for the microbial population to survive and replicate, or may be due to the formation of biofilms attached to the walls or to material which provides some protection against the harsh environmental conditions. 

The presence of a *Variovorax* strain in abundance during the reactor operation might be related to its ability to utilize dihydrogen produced during the working cycle. High energy radiations emitted by the burning fuel generate enormous amounts of dihydrogen through water radiolysis. Some strains of *Variovorax* (biotype I) are lithoautotrophic and are capable of growth using dihydrogen as an energy source [27,28]. The advantage conferred to *Variovorax* by this capacity might explain its prevalence over other genera unable to use dihydrogen energy. Noteworthy, the genus *Variovorax* has been detected in radiative environments, such as in a uranium contaminated soil in China [29] and in the spent fuel pool of the Cofrentes nuclear power plant, Spain, where a *Variovorax paradoxus* strain has been identified after a cultivation step [2]. To counter the radiative and metallic stress, *Variovorax* has been shown to possess multiple metal resistance elements as well as elements involved in antioxidant response [30]. All these specificities, combined to its capacity to use dihydrogen, makes this genus well equipped to survive in the radiative, oligotrophic and metallic conditions which prevail in nuclear pools. Although the *Variovorax* genus has great adaptability and survival capabilities to various environmental conditions, when the reactor shuts down, the dihydrogen levels dropped, and other genera not sufficiently competitive during the reactor operation might have been better adapted to the new conditions. This may explain the disappearance of *Variovorax* to the benefit of one *Methylobacterium* species. In further experiments, it would be interesting to test the ability of *Methylobacterium* to grow with *Variovorax* when co-cultured and describe their relationships.

**Figure 3:**
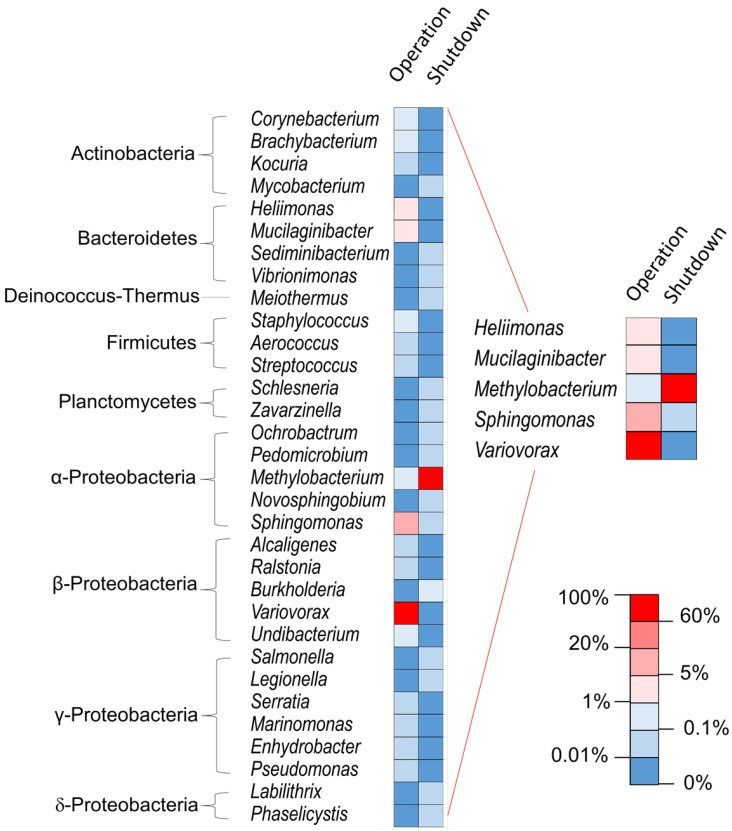
Comparison of the microbial profile at the taxonomic level genus at point A depending on the functioning mode, analyzed by metabarcoding. Mean taxon abundance obtained by the different methods (see Materials and Methods Section).

The second main genus during operation, *Sphingomonas*, remained present at shutdown essentially at points of higher dose rates. This species might be less competitive in an oligotrophic environment when the gamma radiation pressure alleviates. The dramatic alterations in the microbial profile depending on the functioning mode (Figure 3) is likely due mainly to variation in radiation doses and in water characteristics, including dihydrogen and radionuclide concentration. Going forward, it will be worthwhile to characterize how these microbial communities function by means of metaproteomics. In the present work, tandem mass spectrometry data were used for the identification of the taxa but the number of peptides detected were lower than in a metaproteomics classical approach where the biological material is more abundant. In order to exploit the data at protein level, it will be necessary to increase the quantity of biological material to analyze, and thus increase the volume to be sampled while maintaining the safety conditions associated with the handling of radioactive samples. Of note, the genera *Methylobacterium*, *Sphingomonas*, and *Variovorax* have been identified in SNFPs in Spain and in the USA [1,2,22] and some *Methylobacterium* and *Streptomyces* were found in soils contaminated by radionuclides [31,32]. Several species of these last two genera exhibit strong gamma-radiation resistance [32,33].

## 4. Conclusions

Against all odds, we detected the presence of diverse microorganisms in the extreme environment of a working nuclear core pool. This finding breaks new ground in the discovery of radioresistant species and in the understanding of the radiotolerance mechanisms that could be exploited in medicine, in radionuclide bioremediation in the environment or in nuclear installations, and in space programs. The identification of the dominant genus *Variovorax* during operation calls for the search for other hydrogen-metabolizing microorganisms in functioning nuclear facilities. Finally, it should be noted that the strategy combining proteotyping by tandem mass spectrometry and 16S rRNA amplicon sequencing proved worthy to decipher the microbiota composition in such samples and could be more largely exploited in environmental microbiome analysis.

## Data Availability

Sequence data generated in this study has been made available at the Sequence Read Archive (SRA) on NCBI under project number PRJNA679180.

## Supplementary Materials

The following are available online at https://www.mdpi.com/2076-2607/8/12/1857/s1, Table S1: Content in gamma-emitter radionuclides in the reactor water; Table S2: Analysis of the sequencing data obtained for the metabarcoding analysis of water sampled from the reactor’s cooling pool based on 16S rRNA amplicon sequencing, Figure S1: Rarefaction curves; Figure S2: Microbial communities present in the cooling pool of the reactor’s core during operation analyzed at Loc. A, analyzed by 16S rRNA amplicon sequencing; Figure S3: Microbial communities present in the cooling pool of the reactor’s core during shutdown at Loc. A, Figure S4: at Loc. B, and Figure S5: at Loc. C, analyzed by 16S rRNA amplicon sequencing; Figure S6: Comparison of microbial communities present in the pool water at shutdown depending on the sampling point; Table S3: Microbial communities determined by direct proteotyping; Table S4: Challenging potential contamination during sample pretreatment and 16S rRNA amplicon sequencing analyses and validation of taxa genuine presence; Table S5: Determination of a cut-off threshold for the analysis of Illumina MiSeq sequencing data using a ZymoBIOMICS microbial community DNA standard; Supplementary methods.
