# Supplementary material for: Direct Meta-Analyses Reveal Unexpected Microbial Life in the Highly Radioactive Water of an Operating Nuclear Reactor Core
Source: Microorganisms. 2020 Nov 25;8(12):1857. doi: 10.3390/microorganisms8121857 (PMC7760952; doi:10.3390/microorganisms8121857)
Supplement: Supplementary file 1 [file microorganisms-08-01857-s001.pdf]

Direct meta-analyses reveal unexpected microbial life in the highly radioactive water of an operating nuclear reactor core

Pauline C. M. Petit, Olivier Pible, Valérie Van Eesbeeck, Claude Alban, Gérard Steinmetz, Mohamed Mysara, Pieter Monsieurs, Jean Armengaud and Corinne Rivasseau

SUPPLEMENTARY MATERIALS

Supplementary materials include:

• Supplementary data 1. Content in gamma-emitters radionuclides in the reactor water (Table S1).

• Supplementary data 2. Analysis of water sampled from the cooling pool of the reactor's core assessed by metabarcoding based on 16S rRNA amplicon sequencing including:
- Analysis of the sequencing data (Table S2)
- Rarefaction curves (Figure S1),
- Microbial communities present in the cooling pool of the reactor's core during operation analyzed at Loc. A (Figure S2),
- Analysis of microbial communities present in the cooling pool of the reactor's core during shutdown at Loc. A (Figure S3), at Loc. B (Figure S4), and at Loc. C (Figure S5),
- Comparison of microbial communities present in the pool water at shutdown depending on the sampling point (Figure S6);

• Supplementary data 3. Microbial communities determined by direct proteotyping by tandem mass spectrometry (Table S3);

• Supplementary data 4. Challenging potential contamination during sampling, pretreatment and 16S rRNA amplicon sequencing analyses and validation of taxa presence (Table S4);

• Supplementary data 5. Determination of a cut-off threshold for the analysis of Illumina MiSeq sequencing data using a ZymoBIOMICS microbial community DNA standard (Table S5);

• Supplementary methods.

SUPPLEMENTARY DATA 1. Content in gamma-emitters radionuclides in the reactor water.

Table S1. Nature and concentration of the gamma-emitters radionuclides present in the reactor water in operation and at shutdown.

Isotope	Activity (Bq/m^3)	
	Operation	Shutdown
^{24}Na	2.9×10^9	
^{99m}Tc	9.0×10^7	2.5×10^5
^{188}Re	7.1×10^7	
^{7}Be	4.0×10^7	
^{187}W	2.7×10^7	
^{41}Ar	1.7×10^7	
^{197m}Hg	1.3×10^7	
^{56}Mn	1.1×10^7	
^{186}Re	6.5×10^6	3.7×10^5
^{99}Mo	2.6×10^6	1.7×10^5
^{51}Cr	2.1×10^6	1.6×10^6
^{122}Sb	3.4×10^6	1.7×10^5
^{124}Sb		2.8×10^5
^{54}Mn	3.7×10^5	3.6×10^4
^{58}Co		2.5×10^5
^{60}Co		1.3×10^5

SUPPLEMENTARY DATA 2. Analysis of water sampled from the cooling pool of the reactor's core assessed by metabarcoding using 16S rRNA amplicon sequencing

Table S2. Sequencing data analysis of water sampled from the nuclear core's pool. 16S rRNA amplicon sequencing results and diversity obtained by means of Illumina MiSeq sequencing of water samples collected at the three sampling points Loc. A, Loc. B, and Loc. C in the core's reactor cooling pool during operation and at shutdown.

Osiris reactor	Sampling point	Sample preparation	Raw reads	Selected reads	OTUs number	Shannon index
In operation (2015)	Loc. A	Meth. 1	50908	36387	20	1.02
	Loc. A	Meth. 2	53243	37293	17	0.67
At Shutdown (2017)	Loc. A	Meth. 1	134752	71157	30	0.28
	Loc. A	Meth. 2	184589	99970	33	0.22
	Loc. A	Meth. 3	274423	127024	31	0.27
	Loc. B	Meth. 1	7167*	3696*	12	1.22
	Loc. B	Meth. 2	55638	28977	16	0.88
	Loc. B	Meth. 3	181945	95759	30	0.20
	Loc. C	Meth. 1	205579	100394	32	0.21
	Loc. C	Meth. 2	84569	45166	38	0.82
	Loc. C	Meth. 3	193922	105307	35	0.16

The three sampling points Loc. A, Loc. B, and Loc. C correspond respectively to samplings at the pool's bottom, inside the core unit, and above the core unit (see Figure 1a). The three methods Method 1, Method 2, and Method 3 used to prepare water samples prior to amplicon sequencing consisted respectively in centrifugation followed by phenol-chloroform-isoamyl alcohol DNA extraction, filtration followed by phenol-chloroform-isoamyl alcohol DNA extraction, and filtration followed by DNA extraction using the DNeasy PowerWater kit.

*The low sequence number obtained for the analysis of water from Loc. B using Method 1 compared to all other samples might be due either a problem at the centrifugation stage for collecting cells, at the DNA extraction stage, or at the sequencing stage. Because of the unique collected sample and the special conditions for sampling, no repeat could be performed. This sample was not taken into account when subsampling was implemented.

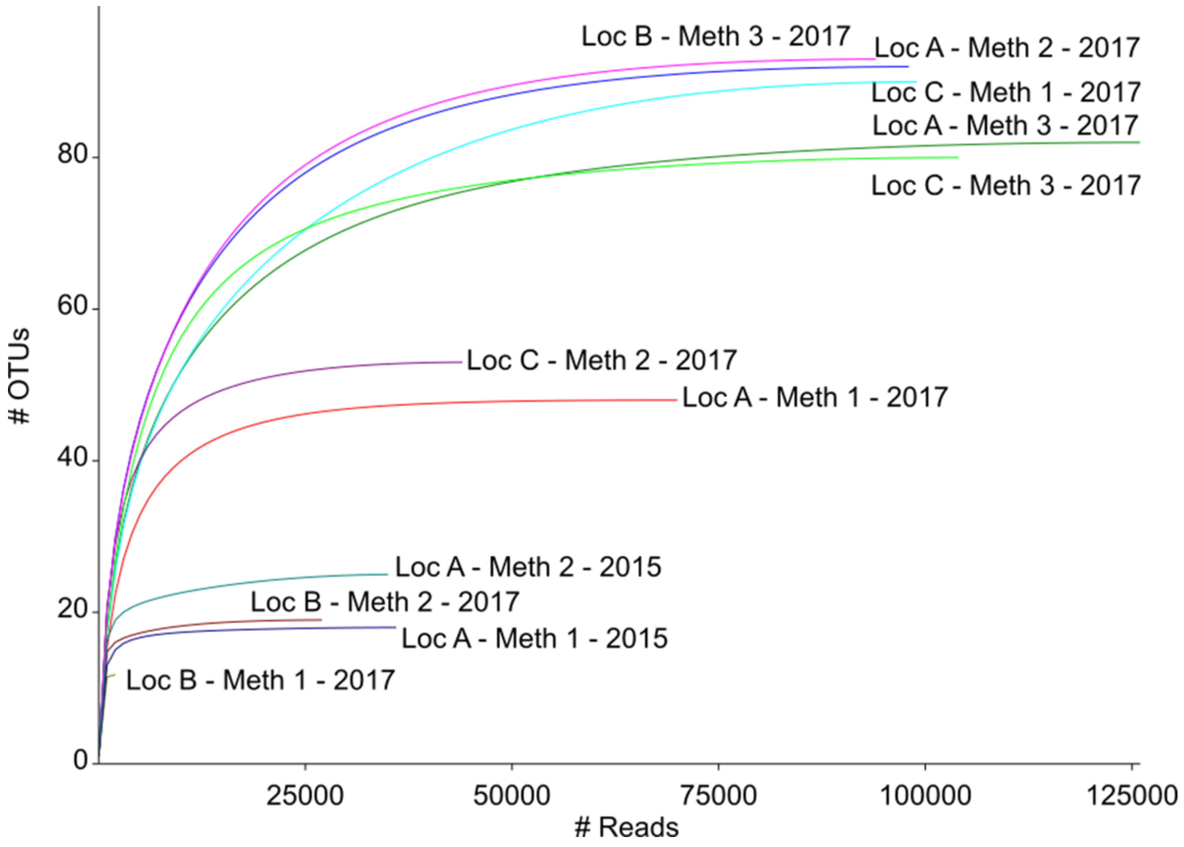

Figure S1. Rarefaction curves of water samples from the reactor's pool. Water samples analyzed by metabarcoding at the three sampling points (Loc. A, Loc. B, and Loc. C, see legend of Table S2) during the 2015 (operation) and the 2017 (shutdown) campaigns after different microorganism concentration and DNA extraction methods (Methods 1, 2, and 3, see legend of Table S2).

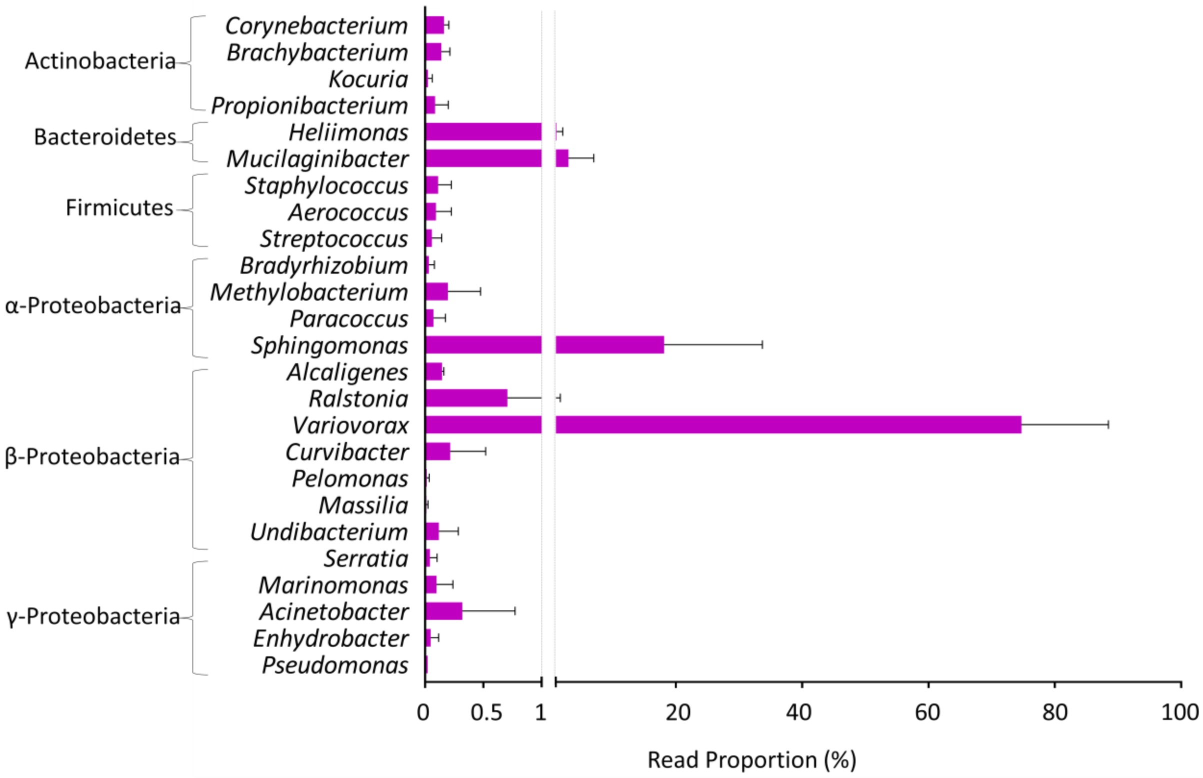

Figure S2. Microbial communities present in the cooling pool of the reactor's core during operation assessed by metabarcoding analysis (16S rRNA amplicon sequencing) from the 2015 samplings at Loc. A (bottom of the pool). OTUs identified up to the taxonomic level genus. The mean and the standard deviation of the sequence abundance obtained by Methods 1 and 2 are shown. Higher taxonomical ranks (phyla or class for the Proteobacteria) are indicated on the left.

Metabarcoding analysis of the core's cooling pool water at shutdown

A total of 44 OTUs corresponding to 20 identified genera were detected in water sampled from Loc. A during shutdown (Figure S3). Some genera included several OTUs. One genus, *Methylobacterium*, and more specifically a single OTU dominated the population, representing 97% of the identified sequences. All other genera represented less than 1% of the sequences. Among the main OTUs identified at low abundance, the *Burkholderia* genus and OTUs belonging to the Chlamydiales order represented 0.45% and 0.5% of the analyzed sequences, respectively. Similarly to the sampling during the reactor operation, a large majority of *Proteobacteria*, which represented 98% of the sequences, was identified during shutdown. Bacteria belonging to other phyla including Acidobacteria, Actinobacteria, Bacteroidetes, Chlamydiae, Deinococcus-Thermus, Firmicutes, and Planctomycetes were detected at low abundance.

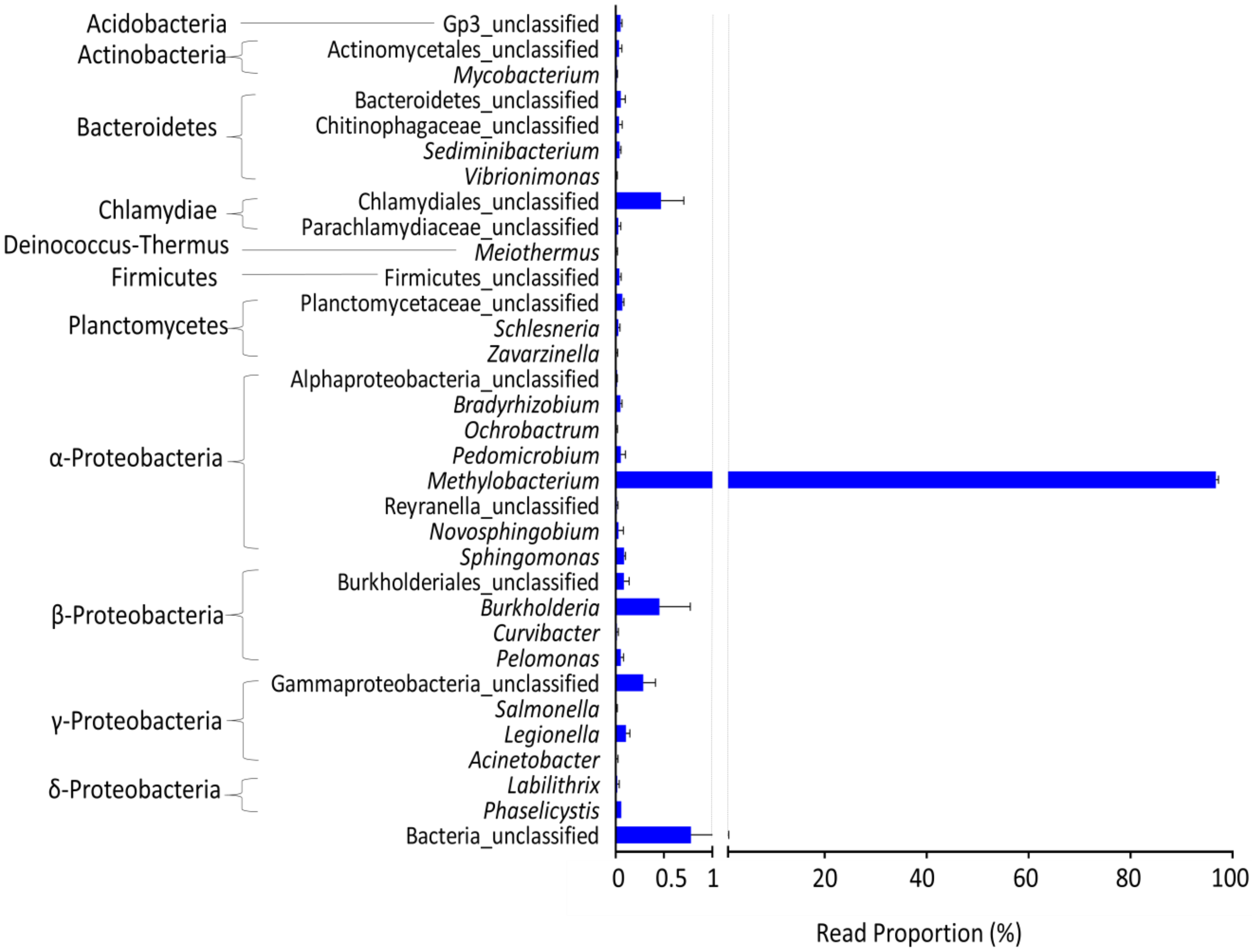

Figure S3. Microbial communities present in the cooling pool of the reactor's core during shutdown assessed by metabarcoding analysis (16S rRNA amplicon sequencing) from the 2017 samplings **at Loc. A** (bottom of the pool). The OTUs were identified up to the taxonomic level genus when possible. The mean and the standard deviation of the sequence abundance obtained by Methods 1, 2, and 3 are shown. Higher taxonomical ranks (phyla or class for the Proteobacteria) are indicated on the left.

Overall 41 OTUs corresponding to 23 identified genera were detected at Loc. B during shutdown (Figure S4). The Proteobacteria phylum represented about 98% of the sequences while Firmicutes represented about 1%. Bacteria belonging to the phyla Acidobacteria, Actinobacteria, Bacteroidetes, Chlamydiae, Deinococcus-Thermus, and Planctomycetes were identified at low abundance (below 0.3%) and low diversity level. In terms of genera, a majority of *Methylobacterium* (90%) was identified, followed by *Sphingomonas* (7%). All other genera were detected in proportion lower than 1%.

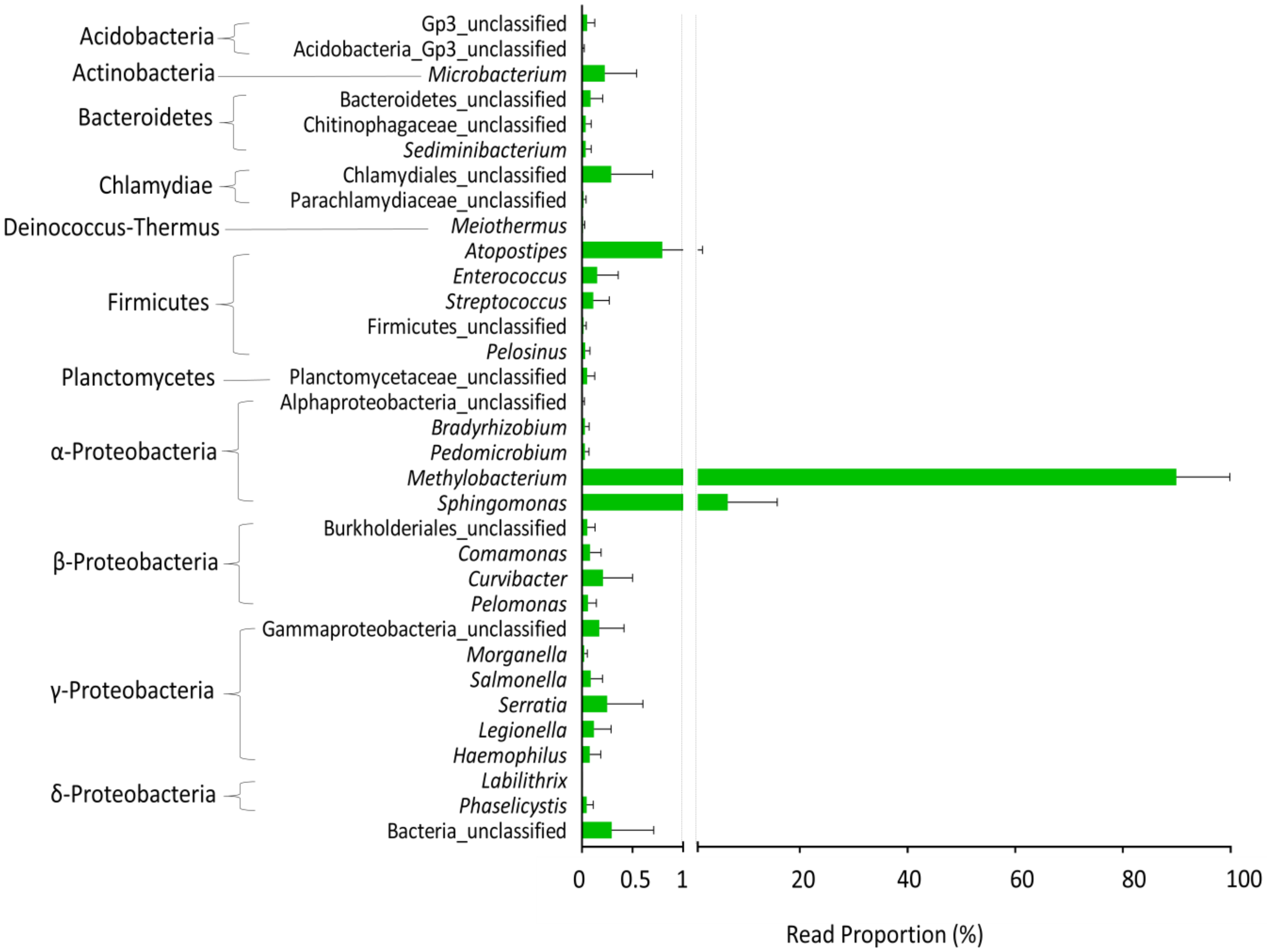

Figure S4. Microbial communities present in the cooling pool of the reactor's core during shutdown assessed by metabarcoding analysis (16S rRNA amplicon sequencing) from the 2017 samplings at **Loc. B** (inside the chimney). Other conditions as in Figure S3 except that mean and standard deviation were calculated from results obtained by Methods 2 and 3.

A total of 67 OTUs corresponding to 34 identified genera were detected at Loc. C (Figure S5). Proteobacteria were predominant, representing 98% of the sequences. At low abundance, bacteria belonging to the phyla Chlamydiae (0.5%), Bacteroidetes (0.3%), and Acidobacteria, Actinobacteria, Deinococcus-Thermus, Firmicutes, Planctomycetes, and Verrucomicrobia (all below 0.1%) were detected. At genus level, *Methylobacterium* dominated the identified sequences, with 87% of the sequences. Another main genus, *Sphingomonas*, was detected in the samples and represented 9% of the sequences. All other genera represented less than 1% of the sequences. Among them, only four were above 0.1%, including the two above-mentioned genera, *Burkholderia*, and *Pelomonas*, which represented about 0.2% of the sequences each. Several OTUs were identified at the level order only and two were above 0.1%, namely "Chlamydiales" and "Rhizobiales", at 0.4% and 0.2%, respectively.

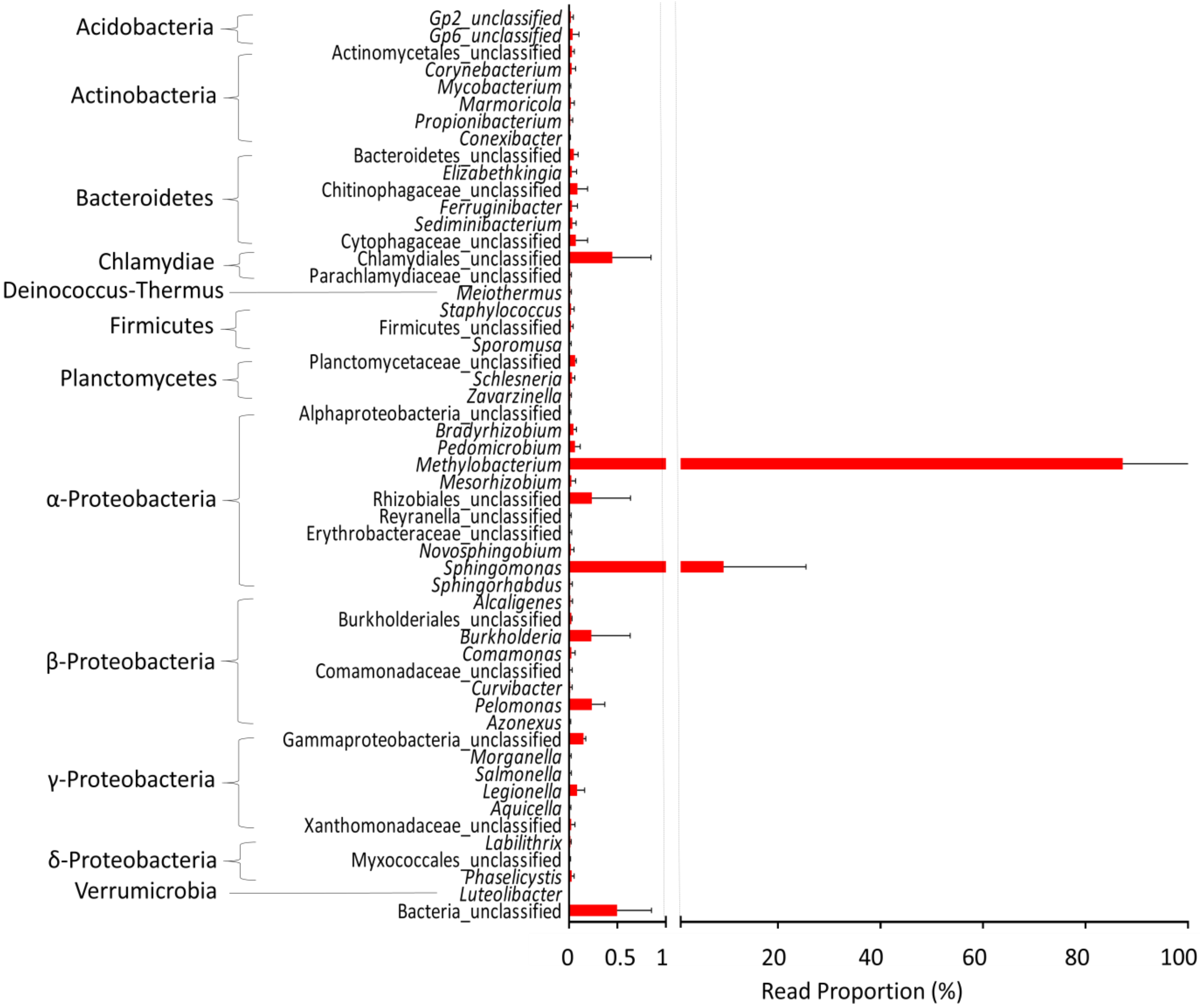

Figure S5. Microbial communities present in the cooling pool of the reactor's core during shutdown assessed by metabarcoding analysis (16S rRNA amplicon sequencing) from the 2017 samplings at **Loc. C** (above the chimney). Other conditions as in Figure S3.

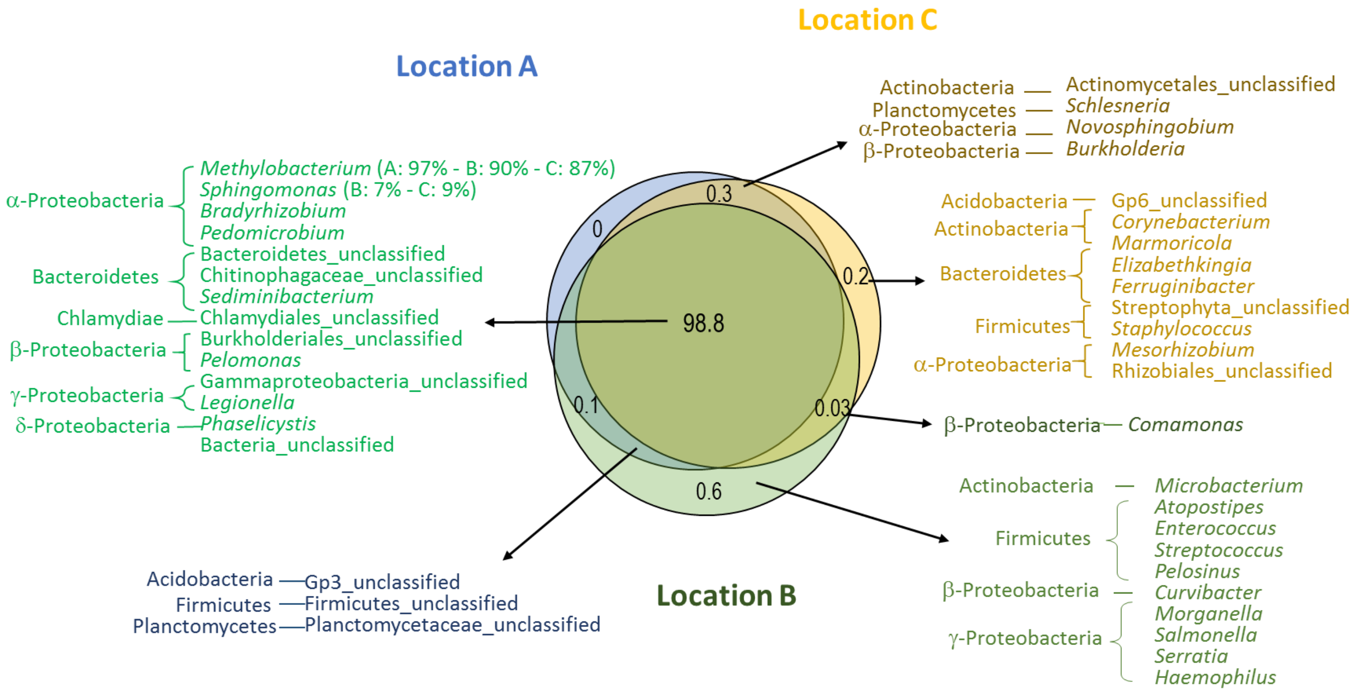

Figure S6. Comparison of microbial communities present in the water of the cooling pool of the reactor's core depending on the sampling point, during shutdown, analyzed by metabarcoding using the 16S rRNA amplicon. Sampling was carried out at three different points (A, B, C) with respective radiation dose rate of below the detection threshold of 0.1 µGy/h, 25 Gy/h, and 15 µGy/h. Metabarcoding data were subsampled at 28,977, identified contaminants were removed, the 0.016% cut-off was applied, and a Venn diagram was drawn to compare the microbial composition at each location. The cumulated relative abundance of all OTUs included in the circles' sectors is indicated on the Venn diagram, in %. The OTUs were identified up to the taxonomic level genus when possible. The relative abundance of each genus at each location is indicated into brackets when it is higher than 1%.

SUPPLEMENTARY DATA 3. Microbiota composition determined by direct proteotyping by tandem mass spectrometry

A direct analysis of peptides from proteins extracted from cells harvested by filtration of 4.5 L of water collected at shutdown was performed by tandem mass spectrometry. While the number of microorganisms present was relatively low for this approach ($12x10^6$, $2x10^6$, and $6x10^6$ cells collected in samples from Loc. A, B, and C, respectively), enough signal could be recorded by shotgun proteomics with 9518, 10964, and 6309 MS/MS spectra assigned to peptide sequences when using a generalist database (NCBInr) at Loc. A, Loc. B, and Loc. C, respectively (Table S3). The taxonomical analysis of this dataset by proteotyping highlighted the presence of two bacterial phyla. At all sampling points, Proteobacteria and Actinobacteria dominated the population with half of the taxon-spectrum matches (TSMs) considered at the phylum taxonomical rank. Based on the identified peptide sequences, four different genera were detected. The three most abundant genera were common to all locations, accounting for at least 97% of the peptide signal. They include i) the Proteobacteria *Methylobacterium* which accounts for 48% of the biomass in average for the three sampling points, and ii) the two Actinobacteria *Asanoa* and *Streptomyces* (25% each in average). A genus belonging to the β-Proteobacteria class, *Pelomonas*, was detected only at Loc. A and Loc. C at 3% and 2% of biomass, respectively (Table S3). Remarkably, this microorganism is not detected in location B while the number of TSMs is higher in this condition, thus indicating that the sensitivity of the approach was not lower for this sample. Based on proteotyping with shotgun MS/MS data, the community profile was not significantly different between all sampling points. Note that MS/MS signal treatments from two blank samples, consisting of sterile LB broth diluted 6X with water on one hand, and sterile LB broth diluted 36X, both returned 0 MS/MS spectra assigned to bacteria. A positive control consisting in a Zymobiomics mixture of ten microorganisms was treated in similar conditions and the results have been recently published [21].

Table S3. Identification of microbial classes and genera present in water of the core's cooling pool by MS/MS proteotyping.

		Location A			Location B			Location C		
Class	Genus	#spePEPs*	#TSMs**	Ratio	#spePEPs*	#TSMs**	Ratio	#spePEPs*	#TSMs**	Ratio
Actinobacteria		515	4802	50%	606	5482	50%	372	3234	51%
	Asanoa	172	4235	25%	205	4839	26%	122	2860	25%
	Streptomyces	121	4044	25%	151	4592	24%	90	2716	25%
Alphaproteobacteria		254	4427	47%	353	5482	50%	180	2982	47%
	Methylobacterium	249	4420	47%	346	5111	50%	174	2976	47%
Betaproteobacteria		158	289	3%	-	-	-	48	93	2%
	Pelomonas	158	289	3%	-	-	-	48	93	2%

*#spePEPs: number of taxon-specific peptides
**#TSMs: number of taxa-to-spectrum matches

SUPPLEMENTARY DATA 4. **Challenging potential contamination during sampling, pretreatment and 16S rRNA amplicon sequencing analyses and validation of taxa presence**

As recommended in sequence-based microbiome studies in order to reduce the impact of contaminants [12, 13], we implemented the following precautions at different sample processing stages, from sample collection to sequencing. Contamination can indeed occur at different stages, whether at the sample collection stage, pre-treatment by centrifugation or filtration, DNA extraction, amplification, library preparation, or sequencing.

- To minimize contamination, we worked with sterile or clean equipment and conditions. Gloves, overshoes, and clean lab coats were worn during all sample preparation steps.

- Water samples were collected in sterile containers either directly connected to a pipe permanently installed and extensively purged before sampling for the sampling at Location A or filled at the sampling point underwater using a Wildco vial at Location B and C.

- Results from the three sampling points at shutdown were similar using either the pipe or the Wildco vial (see below the results of the analysis of molecular variance), thus validating the sampling methodology and indicating that either mode of sampling did not induce unexpected contamination.

- After sample collection, a concentration step by centrifugation or by filtration was carried out in a sterile environment, in order to maximize the starting sample biomass and avoid issues related to low biomass samples in subsequent sample treatment operations. As a consequence, the microbial load in all samples was of the order of 10^6 cells. Salter *et al.* [12] have demonstrated that contamination appears to predominate for microbial amount of 10^3 to 10^4 cells. The microbial amount analyzed in the present study was sufficient to ensure that the microbial profile was dominated by species genuinely present.

- Control tubes containing various culture media (LB, BHI, and TSB diluted ten times in demineralized water, R2A and NB diluted twice in demineralized water) were used to assess potential contamination during this biomass concentration step. They did not show any bacterial growth after one and three months.

- To minimize contamination risk by DNA extraction reagents, two different DNA extraction methods were used, one based on phenol-chloroform-isoamyl alcohol extraction, the other on the DNeasy PowerWater kit from Qiagen (see Methods section). Statistical test (analysis of molecular variance, AMOVA) showed that there was no significant difference between these approaches (p-value 0.252, obtained after subsampling at the lowest read count in order to take all 2017 samples into account). Community profiles were similar regarding the predominant species.

- From the DNA extraction step, samples of different origin and negative control samples were processed alongside the Osiris samples.

- A positive control (Zymobomics DNA standard) was treated alongside the samples from the amplification step to assess possible contamination during 16S rRNA gene sequencing manipulations. The results of this mock sample did not show any contamination at the PCR amplification, library preparation, and sequencing stages. False positives compared to the

nominal composition of this control represented at most 0.004% of the sequences. *Methylobacterium* DNA was detected with 2 reads out of 91,705 reads, corresponding to 0.002% of the reads, well below the 0.016% threshold that we set for significance (Supplementary data 5 and Table S5).

- Analysis of the Zymobiomics DNA standard made it possible to set a significance threshold of 0.016% in order to avoid overestimating an unjustified diversity.

- As mentioned above, Osiris sample microbial profiles were analyzed against experimental samples of other origin, the mock DNA community (Zymobiomics) used as positive control, and highly diluted samples used as negative controls (Samples 1 to 6, Mock DNA sample, and Control samples 1 and 2 in Table S4, respectively). These other samples and the control samples were processed simultaneously with Osiris samples using the same DNA extraction, PCR amplification, library preparation and sequencing protocols and reagents, in order to distinguish sample associated taxa from contaminants. The same reagent batch was used for all 2015 analyses; another unique batch was used for all 2017 analyses. Microbial profile of samples of different origin and of the controls appeared to differ considerably from that of Osiris samples (Table S4).

- In the 2015 run, the control sample 1 presented 122 selected reads, indicating that exogenous contamination introduced during sample processing was very low [12], even though the read number is only roughly indicative of the DNA concentration. Water samples collected from another installation (samples 1 and 2 in Table S4) did not contain any *Variovorax*, which was predominant in Osiris samples. Regarding the other dominant taxon *Sphingomonas*, the corresponding OTU was detected in all samples, but not in the control sample 1. *Sphingomonas* was also isolated after cultivation of the Osiris water samples (see below).

- In the 2017 run, *Methylobacterium* dominated all Osiris samples whereas it was not detected in any samples of different origin (samples 4, 5, and 6 in Table S4) and in the control sample 2. Its presence was confirmed by a non sequence-based method (proteotyping, see below). *Sphingomonas,* present in all Osiris samples, was absent from sample 4 coming from a different facility and from the control sample 2 (Table S4). Conversely, the *Clostridium* taxon, detected in all samples treated in the 2017 run, whatever their origin, and whatever the DNA extraction method used, probably corresponded to a contamination that occurred between the pretreatment stage and the DNA extraction manipulations. Its relative abundance increased conversely with the number of sequencing reads and it became predominant in control sample 2 and in low read number samples (sample 6 in Table S4). The *Proteus* taxon presented a pattern consistent with that of *Clostridium* in samples treated using the phenol-chloroform-isoamyl alcohol DNA extraction method during the 2017 run: high abundance in control sample 2 and relative abundance conversely increasing with decreasing read number. Moreover, it was absent from samples whose DNA was extracted with the DNeasy PowerWater kit, meaning that *Proteus* was probably a contaminant of the phenol-chloroform-isoamyl alcohol DNA extraction method reagents. These genera identified as contaminants were removed from the results.

- A last point that supports the results is that they do not rely only on DNA sequencing analysis but also on identification by proteotyping (protein analysis) and on isolation after cultivation.

- Indeed, the implementation of two completely different and complementary approaches for microbial community analysis, one based on 16S rRNA gene sequencing (metabarcoding), the other on peptide analysis (proteotyping), enabled to confirm the presence of some genera. Both

approaches highlighted the same largely predominant species, *Methylobacterium*, in the 2017 Osiris samples (Figure 2). *Pelomonas* was also identified using both approaches. Taxa confirmed by the two methods cannot be contaminants as the two methods use completely different protocols and reagents, and different type of analyzers. Indeed, their highly complementary facets could be more largely exploited in microbiome analyses.

- Last, the presence of some species was validated by culture-based approaches. *Sphingomonas*, *Ralstonia*, *Kocuria*, *Mycobacterium*, and *Pelomonas* strains were thus isolated from the present Osiris samples after cultivation (data to be published elsewhere and partly published in Hayoun *et al.* [23]).

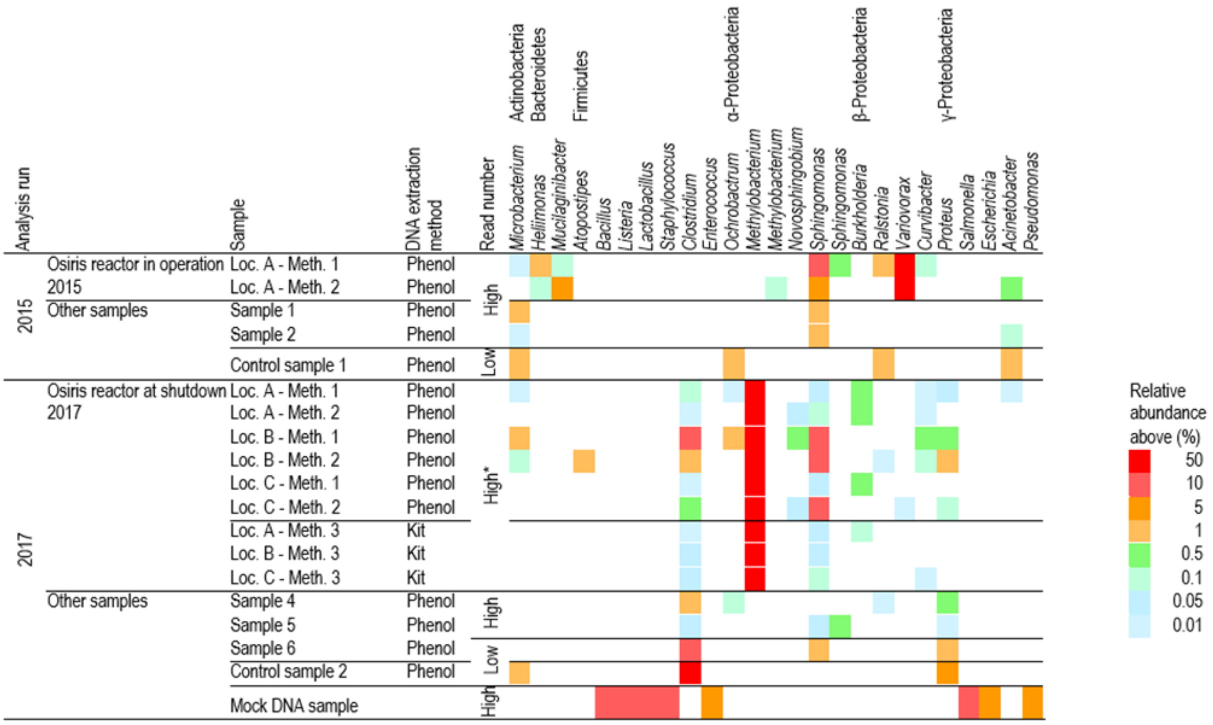

*except in Osiris Loc. B - Meth. 1 sample (see Supplementary Table 1)

Table S4. Taxonomic assignment and relative abundance obtained by 16S rRNA amplicon sequencing in samples from various origin. All 2015 samples on the one hand and all 2017 samples on the other were processed and analyzed concomitantly with the same reagent batch, protocols, and analyzers. In the 2015 run, DNA was extracted using the phenol-chloroform-isoamyl alcohol method (Meth. 1 and Meth. 2). In the 2017 run, DNA was extracted using the phenol-chloroform-isoamyl alcohol method (Meth. 1 and Meth. 2) and the Qiagen DNeasy PowerWater kit (Meth. 3).

SUPPLEMENTARY DATA 5. Determination of a cut-off threshold for the analysis of Illumina MiSeq sequencing data using a ZymoBIOMICS Microbial Community DNA Standard

A microbial community DNA standard (ZymoBIOMICS, Zymo Research) containing genomic DNA extracted from pure cultures of eight bacterial strains was used to establish a cut-off threshold for false-positives rate. This DNA standard (10 µL at a concentration of 10 ng/µL) was amplified and sequenced concomitantly with the experimental water samples using the same reagents and protocol. The sequencing data were analyzed as that obtained from the water samples using the OCToPUS pipeline.

The composition of the DNA standard presented little difference compared to its theoretical composition. The identity of each OTU present in the sample was clearly established and the proportion of their biomasses assessed by the number of sequences was relatively close (within 20%) to the theoretical ones (Table S5). But some sequences belonging to bacteria absent from the mock DNA sample were also identified after sequencing, with a very low number of reads. They are due to false-positives which are easily discriminated as they are in low quantities, representing collectively less than 0.03% of the sequences. Fourteen bacterial sequences could not be clustered with a known OTU. Among these sequences several OTUs were identified as "Bacteria_unclassified". All these OTUs were grouped under the name "Bacteria_unclassified" in Table S5. Among the false-positive sequences identified after sequencing and analysis using OCToPUS, the maximum of sequences clustered into a single OTU was 4 (0.004% of the sequences). Taking into account the number of false-positive bacterial sequences, a 0.016% threshold, corresponding to four times the maximal proportion of the false-positive OTUs, was chosen to analyze data from cooling water microbial samples obtained after the V4V5 Illumina MiSeq sequencing and the OCToPUS analysis.

Table S5. Theoretical proportion of each bacterium in the ZymoBIOMICS DNA standard and proportions obtained after sequencing of the V4V5 region using Illumina MiSeq.

	Theoretical composition	Experimental data		
	Proportion (%)	Number of reads	Proportion (%)	Relative standard deviation to the theoretical composition (%)
**Genera present in the sample**				
Bacillus	15.7	17027	18.6	18.5
Lactobacillus	18.8	16283	17.8	5.3
Staphylococcus	13.3	13483	14.7	10.5
Listeria	15.9	12338	13.5	15.1
Salmonella	11.3	11141	12.1	7.1
Escherichia/Shigella	10.0	9043	9.9	1.0
Enterococcus	10.4	7311	8.0	23.1
Pseudomonas	4.6	5055	5.5	19.6
**Genera not present in the sample**				
Bacteria_unclassified		14	0.015	
Porphyrobacter		4	0.004	
Limnobacter		2	0.002	
Methylobacterium		2	0.002	
Lactobacillales_unclassified		2	0.002	

SUPPLEMENTARY METHODS

DNA extraction. The DNA extraction using a phenol-chloroform-isoamyl alcohol protocol adapted from Vilchez-Vargas *et al.* [14] is detailed here. The sample was centrifuged at 20,000 *g* for 20 min at room temperature (RT). The pellet was resuspended in 1 mL of lysis buffer (100 mM Tris pH 8, 100 mM EDTA pH 8, 100 mM NaCl, 1% polyvinylpyrrolidone, 2% SDS) and disrupted three times by bead-beating at 30 Hz for 60 s in a tube containing 0.1 mm glass beads (Mobio). After centrifugation at 20,000 *g* for 5 min at RT, the supernatant was vigorously mixed with 1 mL of phenol-chloroform-isoamyl alcohol (25:24:1), centrifuged at 17,000 *g* for 1 min at 4°C, then mixed with 700 µL of chloroform, and centrifuged as above. Aliquots of 450 µL were mixed with 45 µL of sodium acetate 3 M and 500 µL of isopropyl alcohol and let overnight at -20°C. After aliquots pooling and centrifugation at 17,000 *g* for 30 min at 4°C, the pellet was washed with 750 µL of 75% ethanol, centrifuged at 17,000 *g* for 5 min at 4°C, dried at 55°C, and dissolved in 60 µL of sterile milliQ water.

Illumina sequencing. The amplification of the target 16S rRNA amplicons was performed by nested PCR using the primer pairs 341F-1061R (5′-CCTACGGGAGGCAGCAG-3′ and 5′-CRRCACGAGCTGACGAC-3′) (20 cycles) + 515YF-926R (5'-GTGYCAGCMGCCGCGGTAA-3' and 5'-CCGYCAATTYMTTTRAGTTT-3') (20 cycles) to target the V4V5 region. The PCR reactions included about 1–10 ng of DNA extract (in a 1 µL volume), 15 pmol of each forward primer and reverse primer (in a 20 µL volume of 1 x MyTaq buffer containing 1.5 units MyTaq DNA polymerase (Bioline)), and 2 µL of BioStabII PCR Enhancer (Sigma). For each sample, a given 10-nt barcode sequence was added to the forward and reverse primers. Each PCR was carried out for 20 cycles using the following parameters: 1 min 96°C pre-denaturation; followed by 20 cycles of denaturation (96°C for 15 s), annealing (50°C for 30 s), and extension (70°C for 90 s). Primers without inline barcodes have been used (341F/1061R) for the first round. For the second round, 1 µL PCR product from the first round was used and the PCR conditions were the same as above. For this reaction barcoded primers have been added (515YF-926R). About 20 ng amplicon DNA of each sample were pooled for up to 48 samples carrying different barcodes. The amplicon pools were purified with one volume AMPure XP beads (Agencourt) to remove primer dimer and other small mispriming products, followed by an additional purification on MiniElute columns (Qiagen). About 100 ng of each purified amplicon pool DNA was used to construct Illumina libraries using the Ovation Rapid DR Multiplex System 1-96 (NuGEN). Illumina libraries were pooled and size selected by preparative gel electrophoresis. Sequencing was done on an Illumina MiSeq using V3 Chemistry (Illumina).
